# Dual channel transformation of scalar and vector terahertz beams along the optical path based on dielectric metasurface

**DOI:** 10.1515/nanoph-2023-0457

**Published:** 2023-09-22

**Authors:** Li Luo, Xiao Liu, Shouxin Duan, Hui Li, Hang Xu, Sui Peng, Bo Liu, Yuting Wang, Lingzhi Wang, Yuxin Zou, Jie Li, Yun Shen, Jianquan Yao

**Affiliations:** Information Materials and Device Applications Key Laboratory of Sichuan Provincial Universities, Chengdu University of Information Technology, Chengdu 610225, China; Department of Physics, School of Physics and Materials Science, Nanchang University, Nanchang 330031, China; Key Laboratory of Opto-Electronics Information Technology (Tianjin University), Ministry of Education, School of Precision Instruments and Opto-Electronics Engineering, Tianjin University, Tianjin 300072, China; Chengdu Advanced Metal Materials Industry Technology Research Institute Limited Company, Chengdu 610300, China

**Keywords:** terahertz, metasurface, longitudinal control, dual channel

## Abstract

The research on terahertz wave manipulation based on metasurfaces has gradually deepened, and the number of functions or electromagnetic control dimensions in a single device is constantly increasing. For the spatial dimension of terahertz field regulation, its design degrees of freedom have been expanded from a single transverse plane to the propagation path. In this paper, we propose a novel circularly polarization multiplexed metasurface for dual channel terahertz wave transmission control. Based on the spatial integration of two heterogeneous meta-atoms, which are spin-decoupled and isotropic, respectively, there are four phase channels that can be used at the same time, thus achieving different switching between vector and scalar beams in different circularly polarization channels along the optical path. For linearly polarized wave incidence, the device exhibits conversion between different vector beams longitudinally. To control more electric field components, we combine focused wavefront design with vector or scalar fields and utilize the focusing induced spin–orbit coupling effect, then complex amplitude switching of longitudinal electric field components is obtained. This article extends the manipulation of terahertz waves along the propagation trajectory based on metasurface from single to dual channel for the first time, providing a reference for the design of multifunctional meta-device in terahertz band.

## 1 Introduction

Metasurfaces comprising micro- and nano-scale components with unique structures represent a novel class of planar optical elements. In contrast to conventional optical devices, metasurfaces enable tailored control over various properties of the optical field, including wavelength, amplitude, phase, and polarization. In recent years, exploiting the capabilities of metasurfaces in optical field manipulation has led to the development of various meta-devices, such as lenses [1, 2], waveplates [3–6], holography generators [7–9], multiplexers [10–12], and polarization converter [13–16]. However, most of these achievements have primarily focused on lateral manipulation of the optical waves, performed within the single plane perpendicular to the propagation direction, such as the focal plane [17–20]. To realize higher-dimensional spatiotemporal control of the optical field and expand its applications, investigations for field control via metasurfaces over the longitudinal dimension of the optical field are imperative. This involves the modulation of electromagnetic characteristics along the direction of light propagation.

Conventional approaches for longitudinal control of the optical field typically involve the cascading of optical devices such as polarizers, waveplates, and spatial light modulators, which necessitates the construction of complex optical paths [21–23]. Initially, in 2012, Chremmos et al. achieved the formation of Bessel-like optical beams propagating along pre-defined trajectories in the far field by modulating the phase of the input wavefront emitted from the input plane [24]. In 2015, Moreno et al. successfully achieved customized polarization distribution along the longitudinal direction of a beam by introducing additional phase delays between two orthogonal polarization components [25]. In 2016, Fu et al. utilized dual spatial light modulators to superimpose vector Bessel beams, generating longitudinally evolving vector fields [26]. These early studies provided rich theoretical support and implementation references for the design of longitudinal characteristics of the light beam. However, these methods still have many shortcomings in terms of optical modulation efficiency, system integration and modulation accuracy [21–30].

Fortunately, metasurfaces exhibit excellent optical characteristics, ultra-thin profiles, and high integration density, offering an effective solution for longitudinal polarization manipulation. In 2020 Fan et al. designed a metasurface that could arbitrarily change the incident circular polarization state and focus it onto a specific focal plane, achieving longitudinal control over the optical field [31]. In 2021, Dorrah et al. developed a metasurface that introduced the Pancharatnam–Berry phase along the propagation direction and controlled the output polarization state by modifying the meta-atoms [32]. In the same year, their team utilized metasurfaces for simultaneous manipulation of polarization and orbital angular momentum along the propagation direction [33]. In 2022, Li et al. proposed a novel approach to manipulating vector beams in the longitudinal direction, enabling simultaneous control over the axial and radial electric field components [34]. Experimental validation in the terahertz regime demonstrated new possibilities for generating vector beams with metasurface. Also, in the same year, Zhang et al. designed a metasurface that achieved simultaneous control of optical fields over the transverse and longitudinal directions, extending polarization optics from two-dimensional to three-dimensional spaces [35]. However, current researches primarily focuses on single-channel control of the optical field with metasurfaces, and investigations into metasurfaces enabling dual or multi-channel for longitudinal control have not yet been reported [31–37]. Developing multi-channel longitudinal control metasurfaces will further enhance the capabilities of optical field manipulation and application potential of meta-devices.

In this paper, we integrate two different meta-atoms into a single metasurface device, enabling dual channel switching of the optical field’s polarization state along the longitudinal direction. We exploit the control capabilities of the two structures on the co- and cross-polarization channels of the optical field. When both channels are incidence with different polarization states, they generate circularly polarized vortex beams with opposite angular quantum numbers and different focal points. We obtain vector beams with varying initial polarization angles at different focal planes by introducing an additional phase difference between the transmitted polarization components. The proposed device allows spatial switching between scalar–vector, vector–scalar, and vector–vector beams in different polarization channels.

## 2 Design and method

**Figure 1:**
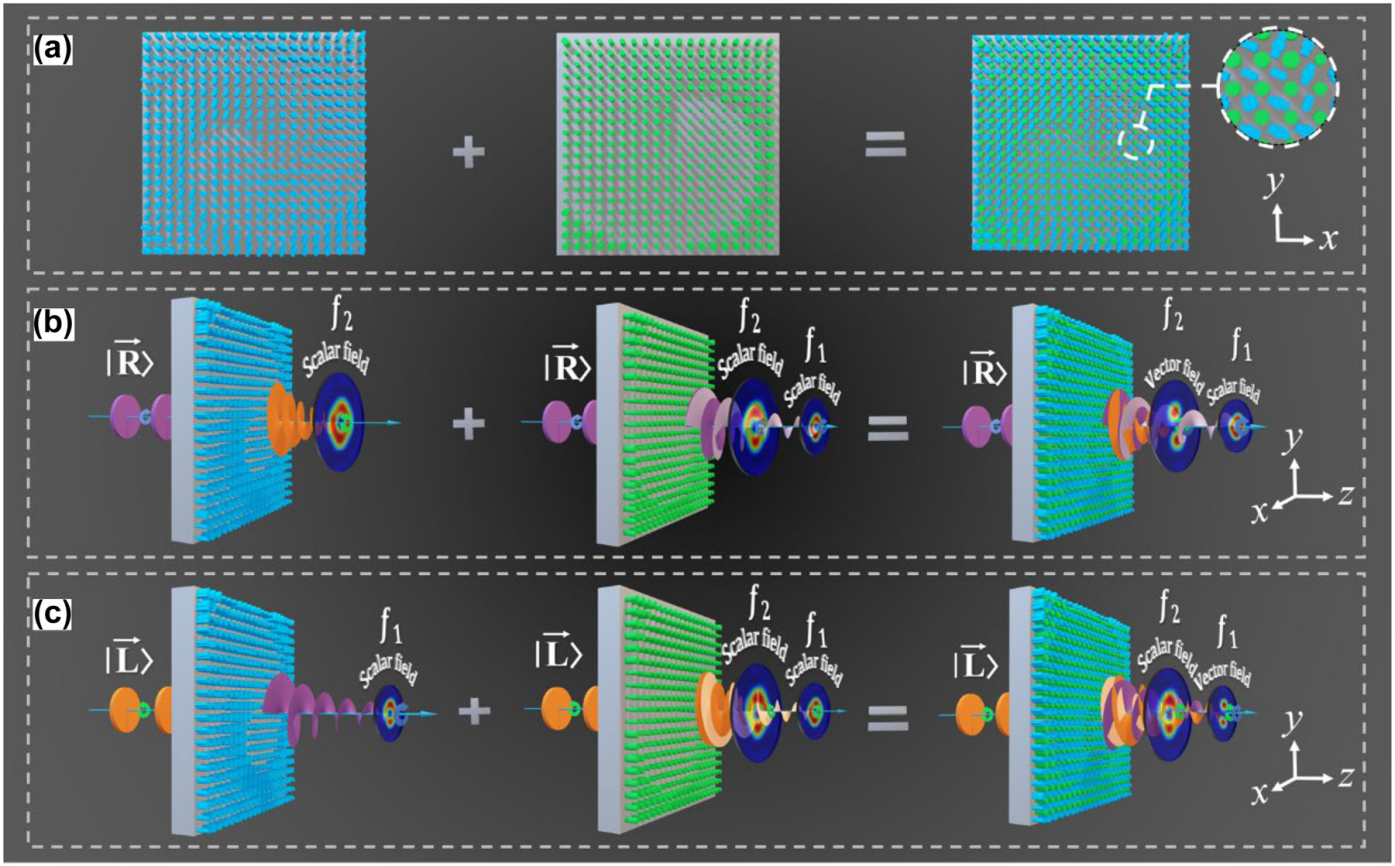
The metasurface with dual channel for longitudinal manipulation of the optical polarization. (a) The arrangement of the two structures on the metasurface, where the first column represents the spin-decoupled unit that controls the cross-polarization component, and the second column represents the isotropic structure that controls the co-polarization component. (b) and (c) The function of the device in left-handed (right-handed) circularly polarized incidence, achieving the switching from vector to scalar (scalar to vector) beam.

We aim to design a metasurface that can independently control the coherent synthesis of the cross- and co-polarization component along the propagation direction to generate vector beams. When the incident light is right- or left-handed circularly polarized (LCP or RCP), the co-polarization channel will generate two vortex beams with the same topological charge number of +1, which is focused at *f*_1_ and *f*_2_, respectively, as shown in Figure 1(b) and (c). Conversely, the cross-polarization channel produces a focused, left-handed (right-handed) circularly polarized vortex beam with an angular quantum number of −1 at *f*_2_(*f*_1_). Precisely, the cross-polarization component is controlled through anisotropic structures, where structure size and rotation direction changes introduce the propagation and geometric phases. On the other hand, the co-polarization component is controlled solely by modifying the size of isotropic structures to introduce the propagation phase. Therefore, the desired manipulation objectives can be achieved simply by introducing the corresponding spatial phase distribution functions into the two channels.

First, we investigate its physical mechanism via the unified Jones matrix and facilitate the analysis of the two units in the proposed metasurface. The Jones matrix for linearly polarized transmission wave of a meta-atom can be written as $T=\left[\begin{array}{cc} t_{xx} & 0\\ 0 & t_{yy} \end{array}\right]$ , where $t_{xx}=T_{xx}{\text{e}}^{\text{i}\varphi_{xx}}$ and $t_{yy}=T_{yy}{\text{e}}^{\text{i}\varphi_{yy}}$ , respectively, denote the transmission coefficients of the component polarized along the *x* and *y* axes (*T*_*xx*_ and *φ*_*xx*_, respectively, refer to amplitude and phase). Assuming the transmission amplitudes of the unit as *T*_*xx*_ = *T*_*yy*_ = 1 and the rotation angle of the structure is *θ*, the linear Jones matrix can be represented as

(1) $$J\left(\theta \right)=\text{M}{\left(\theta \right)}^{T}\times T\times \text{M}\left(\theta \right)$$

where $\text{M}\left(\theta \right)=\left[\begin{array}{cc} \cos\theta & \sin\theta \\ -\sin\theta & \cos\theta \end{array}\right]$ is the rotation matrix. The incident wave is $E_{\text{in}}=\left[\begin{array}{c} \left|\vec{L}\right\rangle \\ \left|\vec{R}\right\rangle \end{array}\right]$ , the linear polarized Jones matrix of the transmitted wave can be written as (see Text S1 for more details in Supplementary Information)

(2) $$\begin{array}{ll} E_{\text{out}} & =J\left(\theta \right)\times E_{\text{in}}\\ & =\frac{1}{2}\left({\text{e}}^{\text{i}\cdot \varphi_{xx}}+{\text{e}}^{\text{i}\cdot \varphi_{yy}}\right)\left[\begin{array}{c} \left|\vec{L}\right\rangle \\ \left|\vec{R}\right\rangle \end{array}\right]+\frac{1}{2}\left({\text{e}}^{\text{i}\cdot \varphi_{xx}}-{\text{e}}^{\text{i}\cdot \varphi_{yy}}\right)\\ & \;\times \left[\begin{array}{c} {\text{e}}^{\text{i}\cdot 2\theta }\cdot \left|\vec{R}\right\rangle \\ {\text{e}}^{-\text{i}\cdot 2\theta }\cdot \left|\vec{L}\right\rangle \end{array}\right] \end{array}$$

Thus, it can be observed that the incident light preserves its original polarization state without any changes when transmitted through the co-polarization channel. Conversely, when transmitted through the cross-polarization channel, it converts into an opposite-handed polarization state, accompanied by the introduction of a geometric phase factor e^±i2*θ*^.

For the structures with a function of spin decoupling, by varying the dimension of the anisotropic units such that the phase difference is Δ*φ*_1_ = *φ*_*xx*_ − *φ*_*yy*_ = *π*, it is possible to eliminate the co-polarization component in the transmitted field. Disregarding the co-polarization component, the transmitted field of the spin decoupling structure, when subjected to the incident field *E*_in_ can be expressed as

(3) $$\begin{array}{ll} E_{\text{cross}} & =J_{\text{cross}}\left(\theta \right)\times E_{\text{in}}\\ & =\left[\begin{array}{c} T_{\text{LR}}\cdot \left|\vec{L}\right\rangle \\ T_{\text{RL}}\cdot \left|\vec{R}\right\rangle \end{array}\right]=\left[\begin{array}{c} {\text{e}}^{\text{i}\left(\varphi_{xx}+2\theta \right)}\cdot \left|\vec{R}\right\rangle \\ {\text{e}}^{\text{i}\left(\varphi_{yy}-2\theta \right)}\cdot \left|\vec{L}\right\rangle \end{array}\right] \end{array}$$

where *T*_LR_, *T*_RL_, *φ*_LR_, and *φ*_RL_ mean the amplitude and phase of the transmitted field, with the first and second subscript indicating the polarization state of the incident and the transmitted field. By considering the propagation and geometric phases, we can derive the design principles for anisotropic units in the metasurface, as shown in Equations (4a)–(4c) (see Text S2 for more details in Supplementary Information).

(4a) $$\varphi_{xx}=\frac{1}{2}\left(\varphi_{\text{LR}}+\varphi_{\text{RL}}\right)$$

(4b) $$\varphi_{yy}=\frac{1}{2}\left(\varphi_{\text{LR}}+\varphi_{\text{RL}}\right)-\pi$$

(4c) $$\theta =\frac{1}{4}\left(\varphi_{\text{RL}}-\varphi_{\text{LR}}\right)$$

For isotropic structures, the phase delays in the *x*- and *y*-directions are equal, i.e., *φ*_*xx*_ = *φ*_*yy*_. As a result, the transmitted field of the unit only contains the co-polarization component. We only need to consider the propagation phase of the isotropic structure to obtain the design principles, which can be expressed as *φ*_LL_ = *φ*_RR_ = *φ*_*xx*_ = *φ*_*yy*_ (see Text S3 for more details in Supplementary Information). Here, *φ*_LL_ and *φ*_RR_ represent the phase of the transmitted field.

## 3 Results and discussion

**Figure 2:**
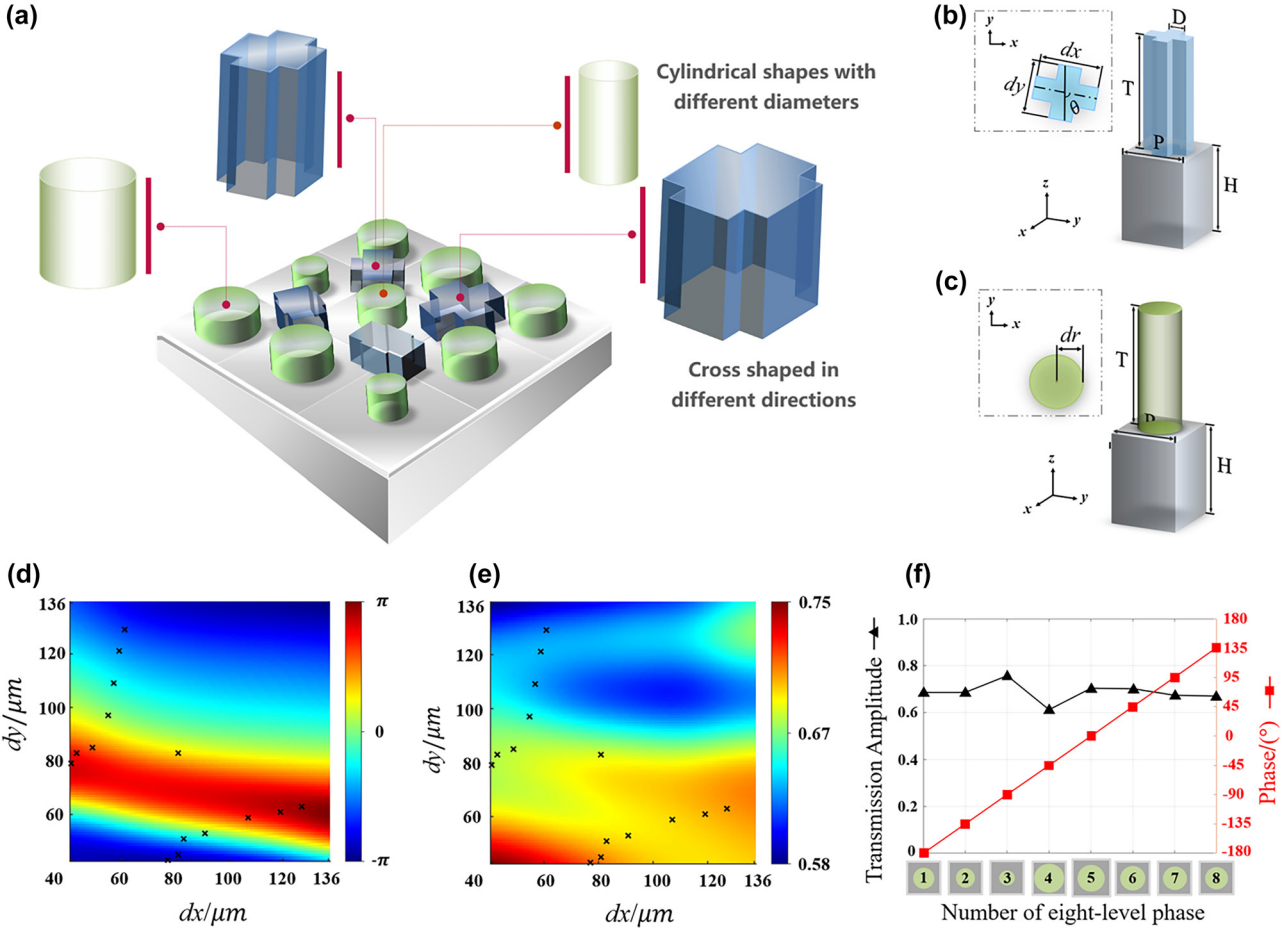
Interleaved arrangement of the two polarization structures on the metasurface, geometric parameters, and the construction of the unit libraries. (a) Interleaved distribution of the anisotropic and isotropic structures. (b) Geometric parameters of the spin-decoupled structure. (c) Geometric parameters of the isotropic structure. (d) and (e) Amplitude and phase of the selected structures from the spin-decoupled units, where the symbols represent the values of d*x* and d*y* for each structure. (f) Amplitude and phase of the selected structures from the isotropic units.

We propose a silicon metasurface composed of heterogeneous meta-atoms to achieve the above functionalities, as shown in Figure 2(a). Figure 2(b) depicts the spin-decoupled structure, consisting of two vertically oriented rectangles and a substrate. Both rectangles have the same width, *D* = 40 μm, while their side lengths are represented by d*x* and d*y*. The period of the structure is *P* = 140 μm. Figure 2(c) shows the isotropic cylindrical pillars with varying radii denoted as d*r*. Both structures have a pillar height of *T* = 200 μm and a substrate thickness of *H* = 300 μm. To analyze the amplitude and propagation phase for *x* and *y* polarization components in different structures, we employed the scanning functionality of the time-domain solver in CST Microwave Studio. There are two changeable variables (d*x* and d*y*) in the spin-decoupled structure, and only d*r* can be changed in the isotropic structure. To achieve complete coverage of the (−*π*, *π*) phase interval, we selected 15 spin-decoupled and 8 isotropic units. In the simulations, the dielectric constant of silicon was set to 11.9, assuming no optical losses. By scanning these parameters, we obtained different amplitudes and propagation phases. To achieve comprehensive control over the phase of the incident wave, we selected the structures that met the desired criteria from the scan results and created separate unit libraries for the two structures. Considering that the air silicon interface generates about 30 % reflection loss, the transmission coefficients of the selected meta-atoms are basically above 0.6, which is already quite efficient. Detailed results can be seen in the Supporting Information (Text S5).

First, we conducted a parameter sweep for d*x* and d*y* within the interval [30 μm, 136 μm], increasing in 1 μm steps, using the CST Microwave Studio. These simulations were performed under a linearly polarized incidence at a 45° angle from the x-coordinate axis to produce Figure 2d and e. Due to the symmetry of the parameters of the *x* and *y* components in the transmitted wave, only the results of *y* polarization are shown here. Then we selected 15 units with a phase gradient of 22.5° to form the parameter library. The amplitudes and phases of each structure are represented by the symbol ‘×’ in Figure 2(d) and (e), respectively.

In cross-polarization channels of anisotropic structures, the phase profiles of vortex beams focused in a single plane are designed separately. When the left-handed circularly polarized light is incident, the metasurface converts the incident light to orbital angular momentum (OAM) states with an azimuthal quantum number of −1 and focuses at the plane of *f*_1_ = 8 mm. When right-handed circularly polarized light is incident, it converts to OAM states with an azimuthal quantum number of −1 and focuses at *f*_2_ = 5 mm. Based on the accumulated optical path distribution of the spiral phase plate and the optical convex lens, the designed phase distribution of the spin-decoupled structure is given by Equations (5a) and (5b)

(5a) $$\varphi_{\text{LR}}=\frac{2\pi }{\lambda }\left(\sqrt{X^{2}+Y^{2}+{f_{1}}^{2}}-f_{1}\right)+l\cdot \tan\frac{Y}{X}$$

(5b) $$\varphi_{\text{RL}}=\frac{2\pi }{\lambda }\left(\sqrt{X^{2}+Y^{2}+{f_{2}}^{2}}-f_{2}\right)+l\cdot \tan\frac{Y}{X}+\Delta \Phi$$

**Figure 3:**
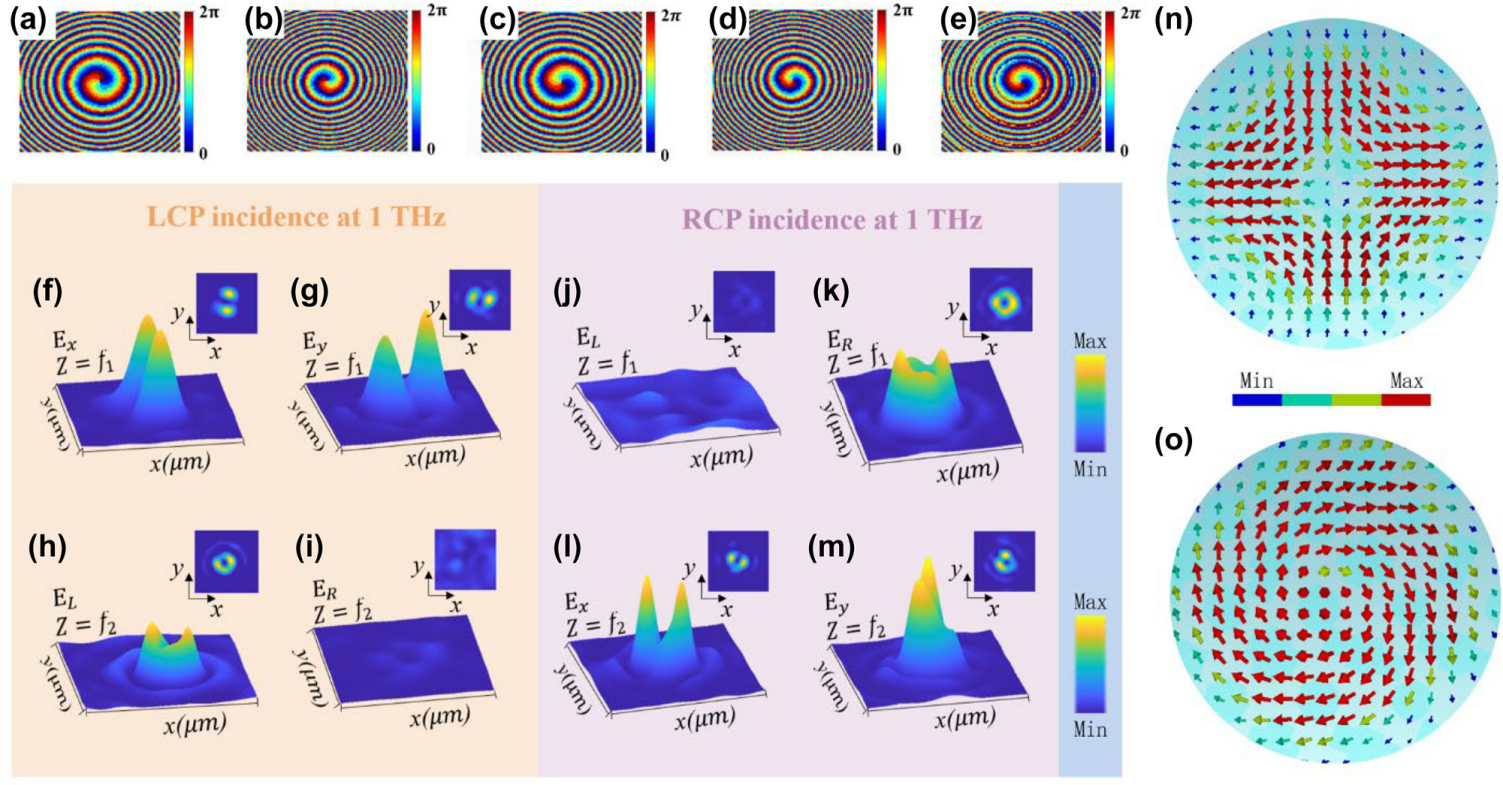
Phase and intensity distributions of the two channels. (a) and (b) Phase distribution for the cross-polarization channel, (c)–(e) Phase distribution for the co-polarization channel. (f)–(i) Electric field intensity distributions when LCP light is incident, focusing the transmitted field onto two focal planes. (f) and (g) Intensity distribution of the transmitted field *E*_*x*_ and *E*_*y*_ at the focal plane *f*_1_. (h) and (i) Intensity distribution of the transmitted field *E*_L_ and *E*_R_ at the focal plane *f*_2_. (j)–(m) Electric field intensity distributions when RCP light is incident, focusing the transmitted field onto the two focal planes. (j) and (k) Intensity distribution of the transmitted field *E*_L_ and *E*_R_ at the focal plane *f*_1_. (l) and (m) Intensity distribution of the transmitted field *E*_*x*_ and *E*_*y*_ at the focal plane *f*_2_. (n) Polarization direction of the electric field at the *f*_1_ focal position when LCP light is incident. (o) Polarization direction of the electric field at the *f*_2_ focal position when RCP light is incident.

Here, *λ* represents the wavelength of the incident wave, (*X*, *Y*) denotes the coordinate of the spatial phase points, and *l* represents the azimuthal quantum number, where *l* = −1 in this case. Figure 3(a) and (b) display the calculated *φ*_LR_ and *φ*_RL_ from Equations (5a) and (5b). An additional phase shift ΔΦ = *π*/2 is introduced to *φ*_RL_, allowing the generation of two vector beams with different initial polarization directions at the two focal planes. Finally, based on Equations (4a)–(4c), spin-decoupling structures satisfy the conditions selected at different positions on the metasurface, thus forming the array of anisotropic elements. Detailed geometric parameters of the structures can be found Supplementary Information Table S1.

We selected eight structures with a 45° phase gradient for the isotropic meta-atoms, as shown in Figure 2(f). Detailed geometric parameters of these structures can be found in Supplementary Information Table S2. The functionality of the co-polarization channel requires that under orthogonal circularly polarized (CP) incidence, the co-polarization structures convert CP light to OAM states with an azimuthal quantum number of +1 and focus them at two focal planes, *f*_1_ = 8 mm and *f*_2_ = 5 mm. The designed phase distribution for these structures is given by Equations (6a)–(6c). Figure 3(c)–(e), respectively, illustrate the computed values of *φ*_LL1_, *φ*_LL2_, and *φ*_LL_.

(6a) $$\varphi_{\text{LL}1}=\varphi_{\text{RR}1}=\frac{2\pi }{\lambda }\left(\sqrt{X^{2}+Y^{2}+{f_{1}}^{2}}-f_{1}\right)+l\cdot \tan\frac{Y}{X}$$

(6b) $$\varphi_{\text{LL}2}=\varphi_{\text{RR}2}=\frac{2\pi }{\lambda }\left(\sqrt{X^{2}+Y^{2}+{f_{2}}^{2}}-f_{2}\right)+l\cdot \tan\frac{Y}{X}$$

(6c) $$\varphi_{\text{LL}}=\varphi_{\text{RR}}=∠\left({\text{e}}^{\text{i}\varphi_{\text{LL}1}}+{\text{e}}^{\text{i}\varphi_{\text{LL}2}}\right)$$

When both structures are interleaved on the same focal plane, the co-polarization channel under LCP or RCP incidence simultaneously generates two vortex beams. Based on the coherent synthesis method of vortex beams, (see Text S4 for more details in Supplementary Information) one of the vortex beams will coherently combine with the cross-polarization component on the same focal plane to form a vector beam, while the other vortex beam will be preserved and focused onto a different focal plane from the vector beam.

The simulation was conducted to verify the process mentioned above. When an LCP beam incidents the metasurface, the coaxial superposition generates a vector beam with a polarization order of +1 and an initial polarization direction *θ*_0_ = 0 at the focal plane *f*_2_ = 5 mm. Figure 3(f) and (g), respectively, depict the electric field intensity of the *x*- (*E*_*x*_) and *y*-axis (*E*_*y*_) polarization components of the vector beam, while Figure 3(n) shows the polarization direction of the electric field at the focal point. It can be observed from the figures that the simulation results align with the theoretical expectations. Simultaneously, a vortex-focusing beam with a topological charge *l*_L_co_ = +1 at the focal plane *f*_1_ = 7.55 mm is generated. Figure 3(h) and (i) demonstrate the left-handed (*E*_L_) and right-handed (*E*_R_) components of the detected output beams, respectively. Only *E*_L_ is present when the input is an LCP beam, indicating the generation of a scalar light field at that focal plane. In addition, it has good focusing performance in the range of 0.8–1.1 THz, accompanied by a slight lens chromatic aberration. (Supplementary Information Text S5).

In the case of RCP beam illumination, the coaxial superposition at the focal plane *f*_2_ generates a vector beam with a polarization order of −1 and an initial polarization direction *θ*_0_ = ΔΦ/2 = *π*/4. Figure 3(l) and (m) display the electric field intensity *E*_*x*_ and *E*_*y*_ of this vector beam, and Figure 3(o) represents the polarization direction of the electric field at the focal point. Additionally, at the focal plane *f*_1_, a vortex-focusing beam with a topological charge *l*_R_co_ = +1 is generated. Figure 3(j) and (k), respectively, exhibit *E*_L_ and *E*_R_ of the detected output beams. Similarly, only *E*_R_ is present when the input is an RCP beam, indicating the generation of a scalar light field at that focal plane.

**Figure 4:**
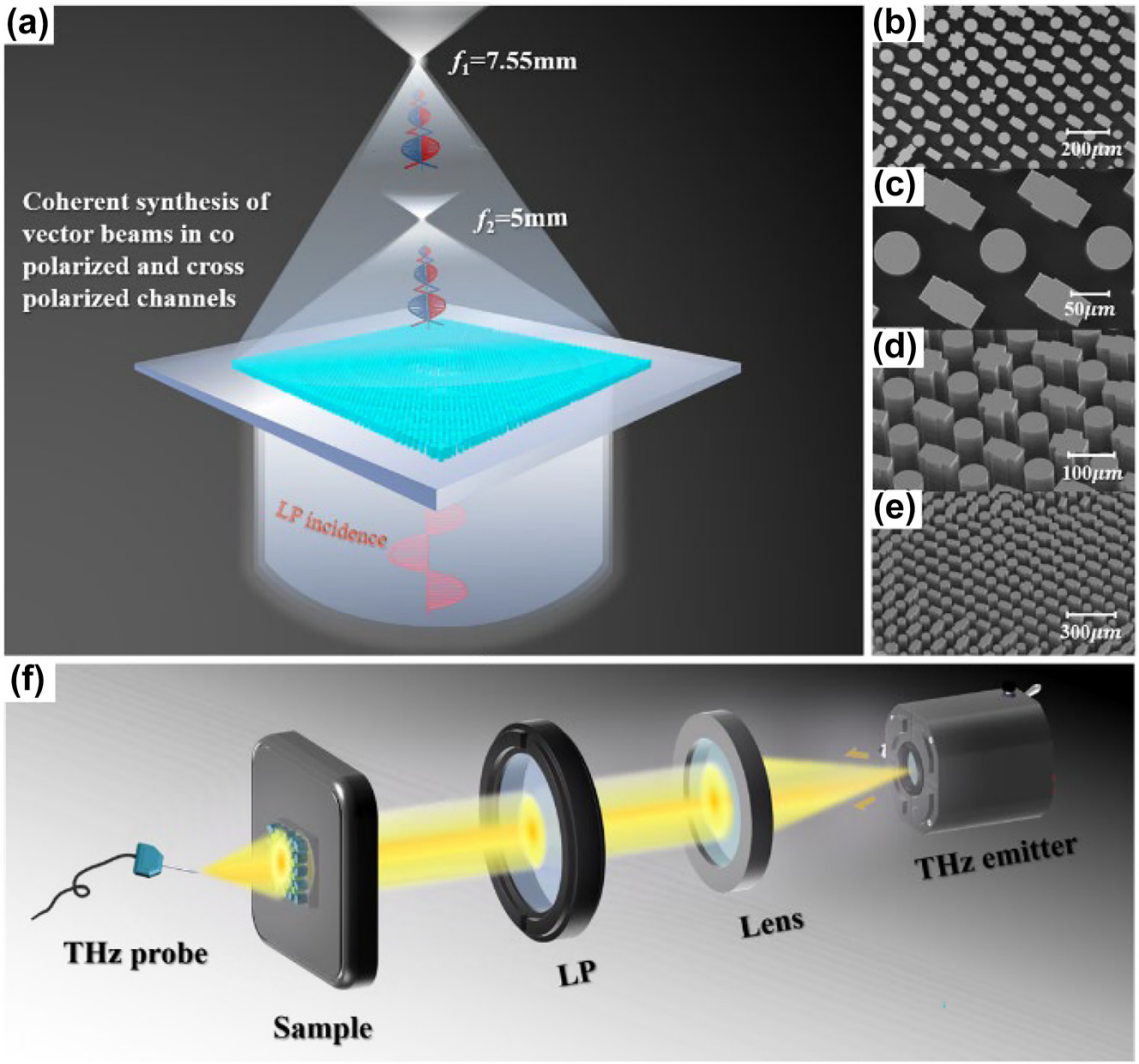
Experimental characterization scheme for the proposed metasurfaces. (a) Schematic of the transmitted field when a linearly polarized beam is incident on the structure. (b)–(e) SEM images of the sample at different angles and magnifications. (f) THz near-field scanning system is equipped with a miniature probe (LP-long-pass filter).

We also fabricated samples to validate the feasibility of our theory. First, the silicon wafer was cleaned, and photoresist was applied. Subsequently, the wafer was exposed through a mask using lithography, developed, and then etched using inductively coupled plasma (ICP). To manipulate both the co- and cross-polarization channels concurrently, we integrated these two structures into a full-silicon metasurface. Figure 4(b)–(e) present the scanning electron microscope (SEM) images of the sample. By introducing a near-field scanning system equipped with a probe, as shown in Figure 4(f), when a THz emitter emits electromagnetic waves at a frequency of 1 THz. After being collimated by a lens, the waves are converted into linearly polarized light using a polarizer before being directed onto the metasurface. Subsequently the vortex beams from both the co-polarization and cross-polarization channels at the focal planes *f*_1_ and *f*_2_ coherently interfere, resulting in two distinct vector beams, as illustrated in Figure 4(a). Finally, a THz probe is utilized to detect the electric field at various positions on the focal plane, yielding experimental data.

**Figure 5:**
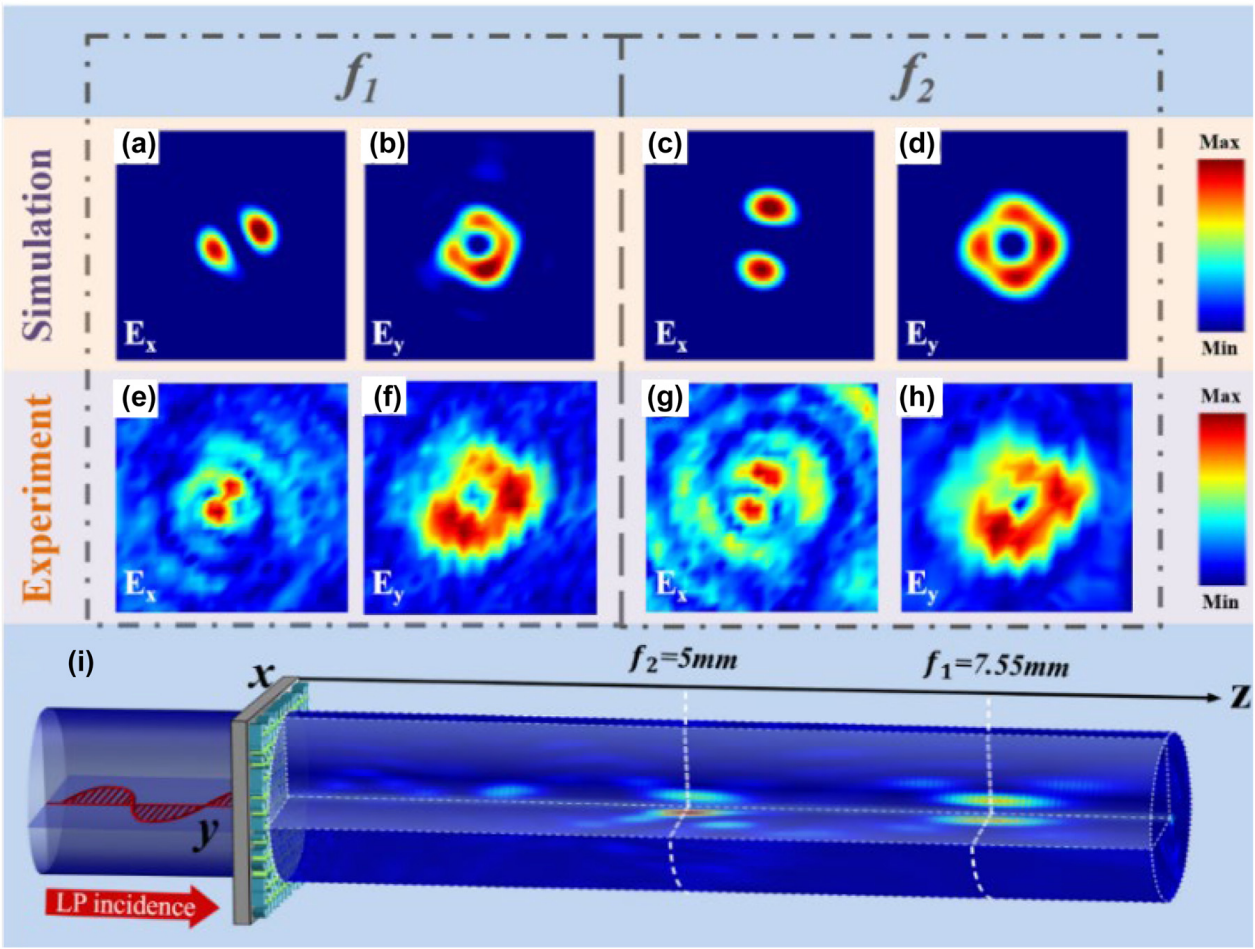
Simulated and experimental results of the transmitted electric field intensity distribution when an LP beam is incident along the *x*-axis. (a)–(d) Simulated results of the transmitted field *E*_*x*_ and *E*_*y*_ at focal point *f*_1_ and *f*_2_. (e)–(h) Experimental results of the transmitted field *E*_*x*_ and *E*_*y*_ at focal point *f*_2_. (i) Schematic diagram of the LP beam incidence.

When we illuminate the metasurface with LP light along the *x*-axis direction, two different vector beams are generated at the focal planes *f*_1_ and *f*_2_, as shown in Figure 5(i). We conducted simulation and experimental verification of the process as mentioned earlier to obtain the intensity distribution of *E*_*x*_ and *E*_*y*_ at different focal points. A comparison between the simulation and experimental results reveals that, apart from the intensity non-uniformity caused by fabrication errors, the experimental results are consistent with the simulation results. It is evident that the intensity of *E*_*x*_ undergoes rotation at focal points *f*_1_ (Figure 5(a)) and *f*_2_ (Figure 5(c)). This is attributed to the addition of a z-coordinate-dependent phase difference (ΔΦ) in the *φ*_RL_ phase during the design of the cross-polarization channel.

Finally, we also analyzed the manipulation performance of the proposed metasurface for intensity and phase distribution of the transmitted electric field along the *z*-axis (*E*_*z*_) via simulation verification. As known, in tight focusing systems, circularly polarized beams transfer a portion of the incident spin angular momentum (SAM) to orbital angular momentum (OAM), generating an axial electric field distribution. Circularly polarized converging beams exhibit spin–orbit coupling effects in the *E*_*z*_ component as [38]

$$l_{\text{longitudinal}}=l_{\text{transverse}}+\sigma$$

where *l*_longitudinal_ represents the longitudinal topological charge, and *l*_transverse_ represents the transverse electric field topological charge, *σ* = ±1 denotes the spin angular momentum.

**Figure 6:**
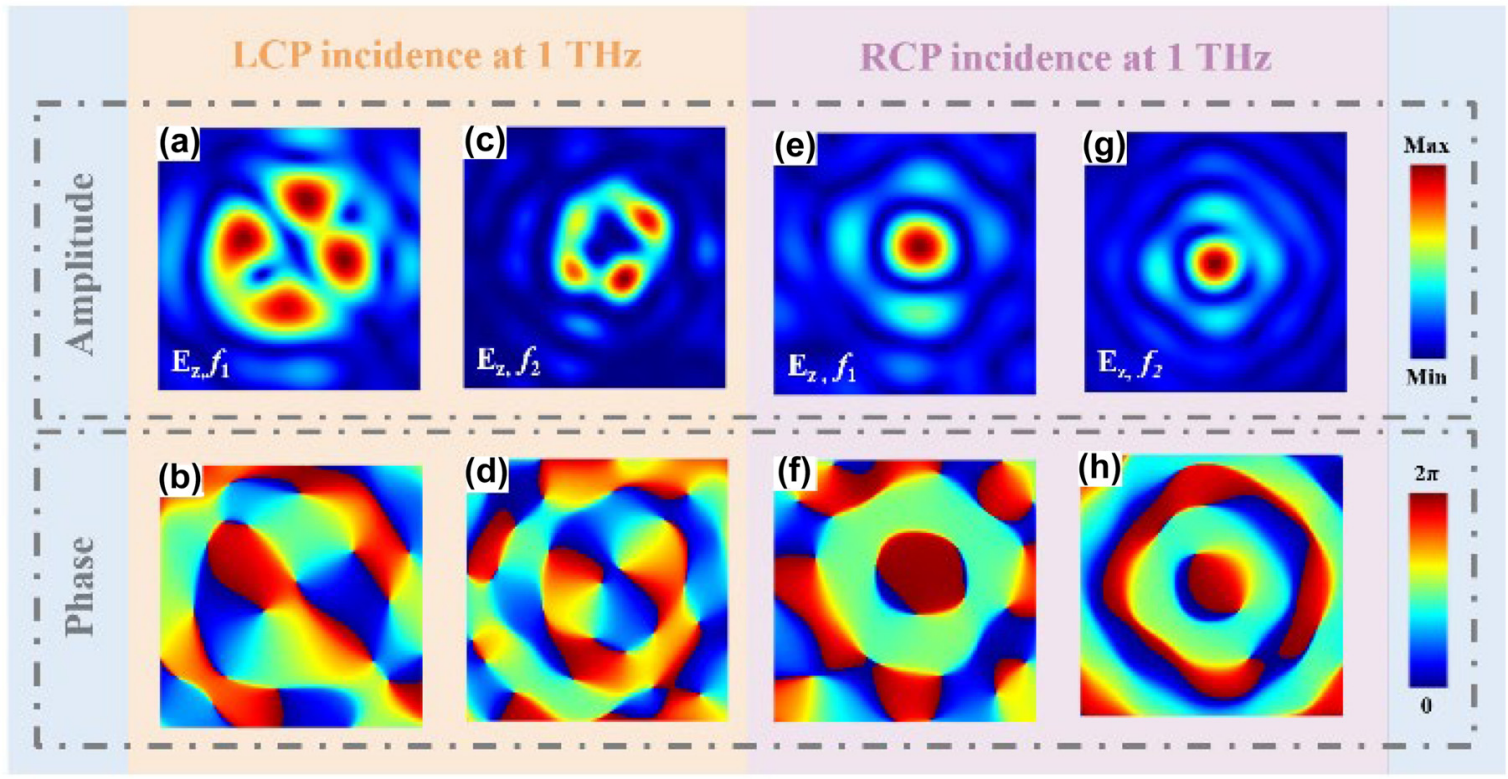
Intensity and phase distribution of the longitudinal electric field component for LCP/RCP incidence. Electric field intensity and phase distribution of the *E*_*z*_ component at focal planes *f*_1_ and *f*_2_ for LCP (a)–(d) and RCP (e)–(h).

From the previous analysis, we know that LCP incidence, at the focal point *f*_1_, the co-polarization component (*l*_transverse_ = +1, *σ* = +1) and the cross-polarization component (*l*_transverse_ = −1, *σ* = −1) form a coherent composite vector beam. Therefore, at the focal plane *f*_1_, the observed *E*_*z*_ field consists of the superposition of the same polarization component with *l*_longitudinal_ = +2 and the cross-polarization component with *l*_longitudinal_ = −2. Figure 6(a) and (b) demonstrate the intensity and phase distribution of the *E*_*z*_ component at that focal point. Furthermore, the co-polarization component also generates a left-handed (*σ* = +1) vortex beam with a topological charge *l*_transverse_ = +1 at the focal point *f*_2_. We will also observe a topological charge *l*_longitudinal_ = +2 of the *E*_*z*_ component at this focal plane. The intensity and phase distribution of the *E*_*z*_ component for this case is depicted in Figure 6(c) and (d).

Under the incidence of RCP, the co-polarization component generates a right-handed (*σ* = −1) vortex beam with a topological charge *l*_transverse_ = +1 at the focal point *f*_1_. At this focal plane, the topological charge of the *E*_*z*_ component *l*_longitudinal_ = 0, as shown in Figure 6(e) and (f). Simultaneously, at the focal point *f*_2_, the co-polarization component (*l*_transverse_ = +1, *σ* = −1) and the cross-polarization component (*l*_transverse_ = −1, *σ* = +1) combine coherently to form a composite vector beam. Consequently, at the observation point *f*_2_, the observed *E*_*z*_ consists of the superposition of the co- and the cross-polarization component with *l*_longitudinal_ = 0. The intensity and phase distribution of the *E*_*z*_ component at this focal point is illustrated in Figure 6(g) and (h).

## 4 Conclusions

In summary, we demonstrated a new scheme for controlling the scalar–vector polarization transformation of terahertz beams in the transmission trajectory. The proposed metasurface has two circularly polarized channels, which achieve different switching of polarization states in the longitudinal direction. We analyzed the unified Jones matrix theory of anisotropic and isotropic units, and a general description method has been given. Then we designed a dual channel metasurface that combines heterogeneous meta-atoms to obtain four phase channels. We use the coherent synthesis method of orthogonal circularly polarized waves to generate vector beams, and the phase difference between components determined the generated polarization state. We conducted simulation and analysis on the dual channel function of the device, demonstrating the vector–scalar or scalar–vector beam switching when different linear/circular polarization waves are incident. We experimentally verified the designed device, showed the intensity distribution of the transmission wave when the samples were irradiated by different linearly polarized terahertz waves, and finally confirmed the working performance of the metasurface. In addition, we also analyzed the specific longitudinal electric field component caused by spin–orbit coupling effect, which also has different complex amplitude distributions on the two focal planes, indicating that the proposed device is suitable for both transverse and longitudinal electric field components. The results of this article are expected to be applied in fields such as high-speed wireless communication, particle acceleration, and terahertz imaging.

## Supplementary Material

Supplementary Material Details

## References

[1] Ni X., Ishii S., Kildishev A. V., Shalaev V. M. (2013). Ultra-thin, planar, babinet-inverted plasmonic metalenses. *Light Sci. Appl.*.

[2] Khorasaninejad M., Zhu A. Y., Roques-Carmes C. (2016). Polarization-insensitive metalenses at visible wavelengths. *Nano Lett*..

[3] Weis P., Paul O., Imhof C., Beigang R., Rahm M. (2009). Strongly birefringent metamaterials as negative index terahertz wave plates. *Appl. Phys. Lett.*.

[4] Zang X., Gong H., Li Z. (2018). Metasurface for multi-channel terahertz beam splitters and polarization rotators. *Appl. Phys. Lett.*.

[5] Xia R., Jing X., Gui X., Tian Y., Hong Z. (2017). Broadband terahertz half-wave plate based on anisotropic polarization conversion metamaterials. *Opt. Mater. Express*.

[6] Sun S., He Q, Xiao S., Xu Q., Li X., Zhou L. (2012). Gradient-index meta-surfaces as a bridge linking propagating waves and surface waves. *Nat. Mater.*.

[7] Li X., Chen L., Li Y. (2016). Multicolor 3D meta-holography by broadband plasmonic modulation. *Sci. Adv.*.

[8] Huang L., Chen X., Mühlenbernd H. (2013). Three-dimensional optical holography using a plasmonic metasurface. *Nat. Commun*..

[9] Ni X., Kildishev A. V., Shalaev V. M. (2013). Metasurface holograms for visible light. *Nat. Commun*..

[10] Zhang K., Chen X., Mühlenbernd H. (2019). High-efficiency metalenses with switchable functionalities in Microwave region. *ACS Appl. Mater. Interfaces*.

[11] Yue Z., Li J., Zheng C. (2022). Manipulation of polarization conversion and multiplexing via all-silicon phase-modulated metasurfaces. *Chin. Opt. Lett.*.

[12] Wang D., Liu F., Liu T. (2021). Efficient generation of complex vectorial optical fields with metasurfaces. *Light: Sci. Appl.*.

[13] Li J., Zheng C., Li J. (2021). Terahertz wavefront shaping with multi-channel polarization conversion based on all-dielectric metasurface. *Photon. Res.*.

[14] Teng S., Zhang Q., Wang H., Liu L., Lv H. (2019). Conversion between polarization states based on a metasurface. *Photon. Res.*.

[15] Li J., Yue Z., Li J. (2022). Wavefront-controllable all-silicon terahertz meta-polarizer. *Sci. China Mater.*.

[16] Chen Y., Zheng X., Zhang X. (2023). Efficient meta-couplers squeezing propagating light into on-chip subwavelength devices in a controllable way. *Nano Lett.*.

[17] Li J., Zheng C., Wang G. (2021). Circular dichroism-like response of terahertz wave caused by phase manipulation via all-silicon metasurface. *Photon. Res.*.

[18] Yuan Y., Sun S., Chen Y. (2020). A fully phase‐modulated metasurface as an energy‐controllable circular polarization router. *Sci. Adv.*.

[19] Wang Z., Yao Y., Pan W. (2022). Bifunctional manipulation of terahertz waves with high-efficiency transmissive dielectric metasurfaces. *Advanced Science*.

[20] Zheng C., Wang G., Li J. (2021). All‐dielectric metasurface for manipulating the superpositions of orbital angular momentum via spin‐decoupling. *Adv. Opt. Mater.*.

[21] Zhu W., Zheng H., Zhong Y., Yu J., Chen Z. (2021). Wave-vector-varying Pancharatnam-Berry phase photonic spin hall effect. *Phys. Rev. Lett.*.

[22] Fu S., Guo C., Liu G. (2019). Spin-orbit optical Hall effect. *Phys. Rev. Lett.*.

[23] Yu N., Genevet P., Kats M. A. (2011). Light propagation with phase discontinuities: generalized laws of reflection and refraction. *Science*.

[24] Chremmos D., Chen Z., Christodoulides D. N., Efremidis N. K. (2012). Bessel-like optical beams with arbitrary trajectories. *Chin. Opt. Lett.*.

[25] Moreno I., Davis J. A., Sánchez-López M. M., Badham K., Cottrell D. M. (2015). Nondiffracting Bessel beams with polarization state that varies with propagation distance. *Chin. Opt. Lett.*.

[26] Fu S., Zhang S., Gao C. (2016). Bessel beams with spatial oscillating polarization. *Sci. Rep.*.

[27] Zhao J., Zhang P., Deng D. (2013). Self-accelerating and self-breathing Bessel-like beams along arbitrary trajectories. *Opt. Lett.*.

[28] He J., Wang X., Hu D. (2013). Generation and evolution of the terahertz vortex beam. *Opt. Express*.

[29] Hao J., Yu Z., Chen Z., Chen H., Ding J. (2014). Shaping of focal field with controllable amplitude, phase, and polarization. *Chin. Opt. Lett.*.

[30] Schulze C., Roux F. S., Dudley A., Rop R., Duparré M., Forbes A. (2015). Accelerated rotation with orbital angular momentum modes. *Phys. Rev. A*.

[31] Fan X., Li P., Guo X. (2020). Axially tailored light field by means of a dielectric metalens. *Phys. Rev. Appl.*.

[32] Dorrah H., Tamagnone M., Rubin N. A., Zaidi A., Capasso F. (2021). Introducing Berry phase gradients along the optical path via propagation-dependent polarization transformations. *Nanophotonics*.

[33] Dorrah H., Rubin N. A., Tamagnone M., Zaidi A., Capasso F. (2021). Structuring total angular momentum of light along the propagation direction with polarization-controlled meta-optics. *Nat. Commun.*.

[34] Li J., Li J., Yue Z. (2022). Structured vector field manipulation of terahertz wave along the propagation direction based on dielectric metasurfaces. *Laser Photon. Res.*.

[35] Zhang F., Pu M., Guo Y. (2022). Synthetic vector optical fields with spatial and temporal tunability. *Sci. China Phys. Mech. Astron.*.

[36] Dorrah H., Rubin N. A., Zaidi A., Tamagnone M., Capasso F. (2021). Metasurface optics for on-demand polarization transformations along the optical path. *Nat. Photonics*.

[37] Liu S., Qi S., Li P. (2021). Analogous optical activity in free space using a single Pancharatnam–Berry phase element. *Laser Photon. Rev.*.

[38] Zhao Y., Edgar J. S., Jeffries G. D., McGloin D., Chiu D. T. (2007). Spin-to-orbital angular momentum conversion in a strongly focused optical beam. *Phys. Rev. Lett.*.

