# Supplementary material for: Dual channel transformation of scalar and vector terahertz beams along the optical path based on dielectric metasurface
Source: Nanophotonics. 2023 Sep 22;12(19):3839–48. doi: 10.1515/nanoph-2023-0457 (PMC11636485; doi:10.1515/nanoph-2023-0457)
Supplement: Supplementary file 1 — Supplementary Material Details [file j_nanoph-2023-0457_suppl_001.docx]

`**Supplementary Information**

**Dual channel transformation of scalar and vector terahertz beams along the optical path based on dielectric metasurface**

Li Luo^1^, Xiao Liu^1^, Shouxin Duan^3^, Hui Li^4^, Hang Xu^4^, Sui Peng^2^, Bo Liu^2^, Yuting Wang^1^, Lingzhi Wang^1^, Yuxin Zou^1^, Jie Li^1,*^, Yun Shen^3^, Jianquan Yao^4^.

^1^ Information Materials and Device Applications Key Laboratory of Sichuan Provincial Universities, Chengdu University of Information Technology, Chengdu 610225, China

^2^ Chengdu Advanced Metal Materials Industry Technology Research Institute Limited Company, Chengdu 610300, China

^3^ Department of Physics, School of Physics and Materials Science, Nanchang University, Nanchang 330031, China

^4^ Key Laboratory of Opto-Electronics Information Technology (Tianjin University), Ministry of Education, School of Precision Instruments and Opto-Electronics Engineering, Tianjin University, Tianjin 300072, China

**Text S1 Transmission Jones Matrix of Metasurfaces**

In this section, we will investigate the physical mechanisms underlying the functionality of metasurface structures based on linear polarization analysis. The linear polarization transmission Jones matrix can be represented as $T=\left(\begin{matrix} t_{\text{xx}} & 0 \\ 0 & t_{\text{yy}} \end{matrix}\right)$. Accordingly, the linear Jones matrix of the metasurface can be expressed as:

Here, $M\text{(}\theta\text{)}=\left(\begin{matrix} \cos\theta& \sin\theta\\ -\sin\theta& \cos\theta\end{matrix}\right)$ represents the rotation matrix, and and represent the transmission coefficients of the electric field propagating along the *x* and *y* axes, respectively. *T*_xx_ and *T*_yy_ denote the amplitudes, while *φ*_xx_ and *φ*_yy_ represent the phases of the electric field along the x and y axes, respectively. The rotation angle of the unit cell structure is denoted as *θ*. By calculation, the expression of Jones matrix can be obtained as

$=\frac{1}{2}\left(t_{\text{xx}}+t_{\text{yy}}\right)\left(\begin{matrix} 1 & 0 \\ 0 & 1 \end{matrix}\right)+\frac{1}{2}\left(t_{\text{xx}}-t_{\text{yy}}\right)\left\{\frac{1}{2}e^{i2\theta}\left(\begin{matrix} 1 & -i \\ -i & -1 \end{matrix}\right)+\frac{1}{2}e^{-i2\theta}\left(\begin{matrix} 1 & i \\ i & -1 \end{matrix}\right)\right\}$ (S1)

Let $\delta^{+}=\left(\begin{matrix} 1 & -\text{i} \\ -i & -1 \end{matrix}\right)$ and $\delta^{-}=\left(\begin{matrix} 1 & i \\ i & -1 \end{matrix}\right)$. We know that$\mathop{|L\rangle}\limits^{\to} =\left(\begin{matrix} 1 \\ i \end{matrix}\right)$ and $\mathop{|R\rangle}\limits^{\to} =\left(\begin{matrix} \text{ }1 \\ -i \end{matrix}\right)$ Therefore, we have ,,,and . Assuming the transmission amplitudes of the unit cell are *T*_xx_=*T*_yy_=1, the transmitted field distribution can be expressed as Equation (S2) when the incident wave is *E*_in_

$$E_{\text{out}}=J(\theta)\times E_{\text{in}}$$

(S2)

$$=E_{\text{out1}}+E_{\text{out2}}$$

According to Equation (S2), we know that *E_out_*_1_ is independent of the rotation angle *θ* of the metasurface structure, and the polarization distribution of the transmitted field is the same as that of the incident field. The rotation angle *θ* of the metasurface structure introduces a geometric phase in *E*_out2_, resulting in a change in the polarization distribution of the transmitted field. When LCP is incident, a geometric phase of 2*θ* is introduced, and the transmitted field becomes RCP. When RCP is incident, a geometric phase of −2*θ* is introduced, and the transmitted field becomes LCP.

The physical mechanism for achieving the desired functionalities is investigated based on circular polarization analysis of metasurface structures. In this scenario, the circular transmission coefficients “co” and “cross” represent the relative circular polarization (CP) states. The Jones matrix of the metasurface can be expressed as：

$$\begin{matrix} J_{\text{circular}}(\theta)=M(\theta)^{\text{T}}\times T_{\text{circular}}\times M(\theta) \\ \text{ }=\left(\begin{matrix} \cos\theta& -\sin\theta\\ \sin\theta& \cos\theta\end{matrix}\right)\left(\begin{matrix} \text{t}_{\text{co}} & \text{t}_{\text{cross}} \\ \text{t}_{\text{cross}} & \text{t}_{\text{co}} \end{matrix}\right)\left(\begin{matrix} \cos\theta& \sin\theta\\ -\sin\theta& \cos\theta\end{matrix}\right) \\ \text{ }=\left(\begin{matrix} \text{t}_{\text{co}}-2\sin\theta\cos\theta\cdot\text{t}_{\text{cross}} & \text{ }\left(\cos^{2}\theta-\sin^{2}\theta\right)\text{t}_{\text{cross}} \\ \left(\cos^{2}\theta-\sin^{2}\theta\right)\text{t}_{\text{cross}} & \text{ t}_{\text{co}}+2\sin\theta\cos\theta\cdot\text{t}_{\text{cross}} \end{matrix}\right) \\ \text{ }=\text{t}_{\text{co}}\left(\begin{matrix} 1 & \text{ }0 \\ 0 & \text{ }1 \end{matrix}\right)+\text{t}_{\text{cross}}\left(\begin{matrix} -\sin2\theta& \text{ }\cos2\theta\\ \cos2\theta& \text{ }\sin2\theta\end{matrix}\right) \end{matrix}$$

$\text{ }=\text{t}_{\text{co}}\left(\begin{matrix} 1 & \text{ }0 \\ 0 & \text{ }1 \end{matrix}\right)+\text{t}_{\text{cross}}\left\{\frac{1}{2}e^{i2\theta}\left(\begin{matrix} i & \text{ }1 \\ 1 & \text{ }-i \end{matrix}\right)+\frac{1}{2}e^{-i2\theta}\left(\begin{matrix} -i & \text{ }1 \\ 1 & \text{ }i \end{matrix}\right)\right\}$ (S3)

Combining equations (S1) and (S3), we obtain:

Where, .

**Text S2 Design Principles of** **Spin-Decoupling Structures**

We require the co-polarization component *t*_co_ to be zero for the spin-coupling metasurface structure to achieve independent polarization distributions in the cross-polarization channel. This can be achieved by introducing a propagation phase through variations in the size of anisotropic meta-atoms, such that the phase difference *∆φ*_1_=*kπ(k*=±1,±2,±3*…)*. We select *∆φ*_1_=*π* to design the spin-coupling structure. In this case, the transmitted field can be represented as:

$$E_{\text{cross}}=T_{\text{cross}}(\theta)\times E_{\text{in}}$$

(S4)

Where *T*_LR_, *T*_RL_, *φ*_LR_, and *φ*_RL_ represent the transmission amplitudes and phases. The first subscript denotes the polarization state of the transmitted wave, and the second subscript indicates the incident polarization. According to Equation (S1), we can infer that in this case *E*_cross_=*E*_out2_.

(S5)

Combining equations Equation (S4) and (S5), we can obtain *φ*_LR_=*φ*_xx_*+*2*θ* and *φ*_RL_=*φ*_xx_−2*θ*. Furthermore, considering the propagation phase difference of the spin-decoupling structure, *∆φ*_1_=*φ*_xx_−*φ*_yy_=*π*, we can derive the design method for anisotropic meta-units on the metasurface.

**Text S3 Design Principles of** **Isotropic Structures**

To achieve independent polarization distribution in the co-polarization channel, it is necessary to set the cross-polarization component *t*_cross_ of the isotropic structure to 0. According to Equation (S1), we can infer that in this case *E*_co_=*E*_out1_. This can be achieved by modifying the dimensions of the anisotropic meta-atoms to introduce a propagation phase and make the phase difference *∆φ*_2_=2*kπ,*(*k*=0, *±*1, *±*2*, …*). In this study, we choose *∆φ*_2_=0 to design an isotropic structure *E*_co_=*E*_out1_. Consequently, the transmitted field can be expressed as follows:

Where *T*_LL_, *T*_RR_, *φ*_LL_, and *φ*_RR_ represent the transmission amplitudes and phase, where the first subscript indicates the polarization state of the transmitted wave and the second indicates the incident polarization.

Based on this, we obtain *φ*_LL_=*φ*_xx_ and *φ*_RR_=*φ*_yy_. Furthermore, according to the phase difference introduced by the spin-decoupling structure, *∆φ*_2_=*φ*_xx_−*φ*_yy_=0, which leads to the design requirement for isotropic metaunits: *φ*_LL_=*φ*_RR_=*φ*_xx_=*φ*_yy_.

**Text S4 Coherent synthesis method of vortex beams for generation of vector beams**

A vector beam with a vortex-type polarization distribution in the cross-section can be expressed as a Jones vector as:

where *A(r)* is the complex amplitude, l is the topological charge (order) of polarization, and *θ*_0_ is the initial polarization direction. The vector beams can be obtained by superposition of orthogonal circularly polarized vortex beams, and let the phase difference between them be Δ*φ* we get:

This means that the initial polarization angle *θ*_0_ of the vector beam is determined by Δ*φ*. If Δφ varies along the transmission direction $\theta_{0}=\frac{1}{2}\Delta\varphi$. It can be seen that the coherent synthesis method produces the longitudinal evolution of the polarization state mainly by designing the longitudinal phase difference of the wave components, and by introducing different phase differences in the two vortex beams, different vector beams can be obtained.

**Text S5 Transmittance and Bandwidth of Metasurface Devices**

We choose two structures in the isotropic and Spin-Decoupling decoupled structure of the metasurface, and use the simulation software to arrange them periodically to show their transmittance in the case of LCP and RCP incidence points, in which the Isotropic Structures structure is shown in Fig. S1 a, with a radius of (22.2 μm), and its transmittance is shown in Fig. S1 c, which shows that the transmittance of the metasurface at 1 THz is about 0.75, which can satisfy the requirements of the design and the suppression of cross-polarization generation is also strongest at this frequency point.

The spin decoupled structure is shown in Fig. S1 b. Its parameters are chosen as *dx*=126 μm and *dy*=60 μm, and its transmittance is also simulated under LCP and RCP incidence, respectively, to obtain Figs. S1 d and e. The transmittance is slightly lower than that of the co-polarization compared to the Isotropic Structures at 1 THz, but it also stays above 0.67, which also meets the design requirements.


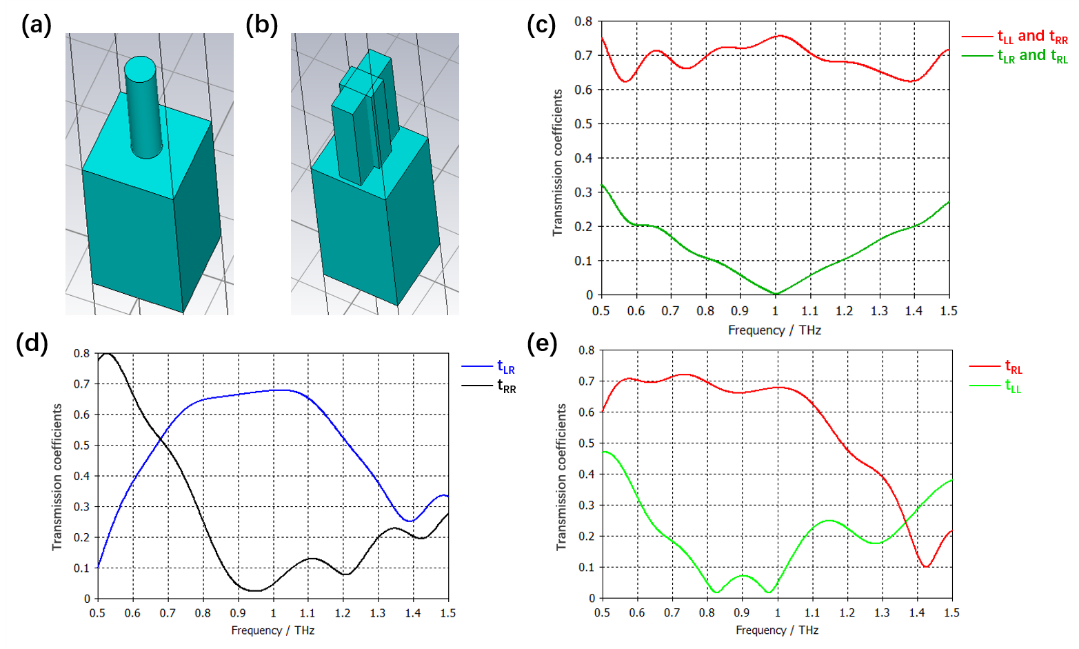


Fig. S1 (a), (b) Structures selected by the simulation software; (c) Transmittance of the isotropic structure at LCP and RCP incidence; (d), (e) Transmittance of the metasurface structure for the spin-decoupled structure at RCP and LCP incidence situations.

We also simulated the metasurface at different frequency points near the operating frequency of 1 THz, and obtained the intensity maps of the electric field along the y-axis of the metasurface at 0.8-1.2 THz (Fig. S2 a-e), and the simulations were performed using the x-polarization incidence to obtain the intensity of the electric field in the y-direction. From the results, it can be seen that experimental results consistent with the theory are obtained at 0.8-1.1 THz.


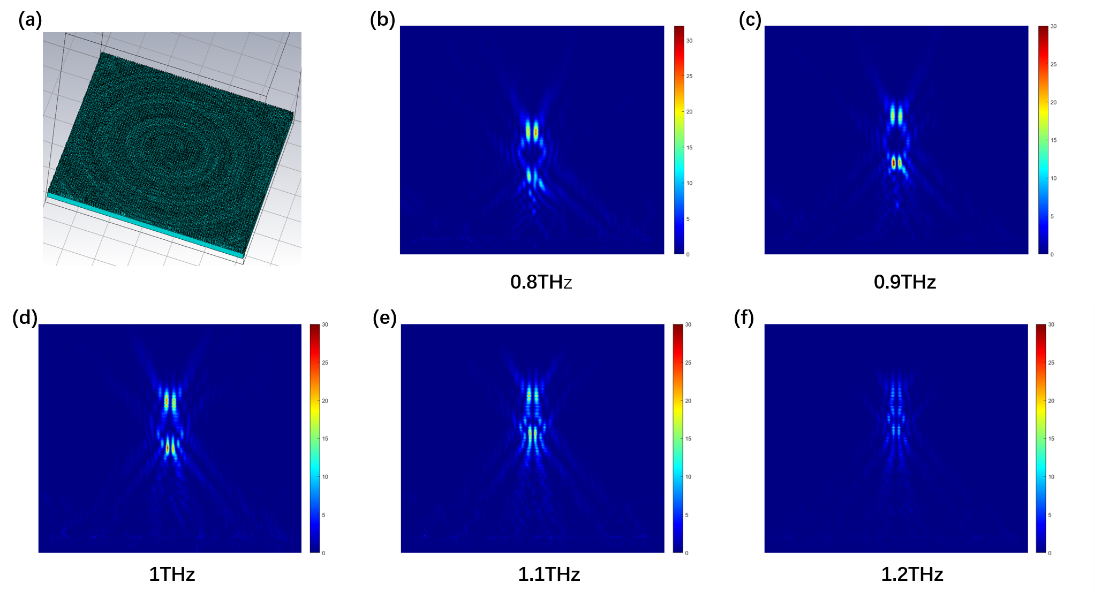


Fig. S2 (a) Metasurface structures; (b)-(f) Ey intensity maps of the metasurface structure for linearly polarized incidence along the x-axis from 0.8 to 1.2 THz

**Table Parameters of Isotropic and Spin-Decoupling Structures**

**Table S1** The spin-decoupling structure with a propagation phase of 180 degrees and a phase gradient of 22.5 degrees.

|  | *dx*(*μm*) | *dy*(*μm*) | *T*_xx_ | *φ*_xx_(°) | *T*_yy_ | *φ*_yy_(°) |
| --- | --- | --- | --- | --- | --- | --- |
| No.1 | 50 | 90 | 0.690 | -172.911 | 0.675 | -5.238 |
| No.2 | 48 | 82 | 0.682 | -155.113 | 0.693 | 25.194 |
| No.3 | 42 | 80 | 0.704 | -133.841 | 0.685 | 46.869 |
| No.4 | 40 | 76 | 0.729 | -123.504 | 0.676 | 74.942 |
| No.5 | 126 | 60 | 0.664 | -82.742 | 0.696 | 89.338 |
| No.6 | 118 | 58 | 0.658 | -63.252 | 0.691 | 108.831 |
| No.7 | 106 | 56 | 0.625 | -43.096 | 0.689 | 132.152 |
| No.8 | 94 | 54 | 0.657 | -20.024 | 0.691 | 156.882 |
| No.9 | 80 | 80 | 0.699 | 1.778 | 0.699 | 1.778 |
| No.10 | 82 | 48 | 0.693 | 25.195 | 0.682 | -155.115 |
| No.11 | 80 | 42 | 0.686 | 46.864 | 0.701 | -133.843 |
| No.12 | 76 | 40 | 0.676 | 74.942 | 0.729 | -123.504 |
| No.13 | 60 | 126 | 0.696 | 89.341 | 0.664 | -82.735 |
| No.14 | 58 | 118 | 0.691 | 108.833 | 0.649 | -63.250 |
| No.15 | 56 | 106 | 0.689 | 132.155 | 0.625 | -43.097 |

**Table S2** The isotropic structure with a propagation phase of 0 degrees and a phase gradient of 45 degrees.

|  | *dr*(*μm*) | *T*_xx_= *T*_yy_ | *φ*_xx_=*φ*_yy_(°) |
| --- | --- | --- | --- |
| No.1 | 30.6 | 0.686 | -179.749 |
| No.2 | 27.8 | 0.686 | -134.852 |
| No.3 | 22.2 | 0.757 | -89.728 |
| No.4 | 42.6 | 0.612 | -45.008 |
| No.5 | 38.7 | 0.704 | -0.194 |
| No.6 | 36.1 | 0.702 | 45.224 |
| No.7 | 34.1 | 0.674 | 90.347 |
| No.8 | 32.4 | 0.671 | 135.827 |
